# 我国单倍体造血干细胞移植的现状和未来

**DOI:** 10.3760/cma.j.cn121090-20240215-00061

**Published:** 2024-07

**Authors:** 晓冬 莫, 晓军 黄

**Affiliations:** 北京大学人民医院，北京大学血液病研究所，国家血液系统疾病临床医学研究中心，造血干细胞移植治疗血液病北京市重点实验室，北京 100044 Peking University People's Hospital & Peking University Institute of Hematology, National Clinical Research Center for Hematologic Disease, Beijing Key Laboratory of Hematopoietic Stem Cell Transplantation, Beijing 100044, China

## Abstract

供者缺乏是造血干细胞移植广泛使用的最大障碍。新型移植方案的建立、完善使得单倍体造血干细胞移植成为临床常规，大量血液病患者得以从中获益，单倍体供者已经成为中国异基因造血干细胞移植最重要的供者来源。本文聚焦单倍体造血干细胞移植在我国开展的现状及未来发展趋势，增加临床医师对单倍体造血干细胞移植的了解。

## Abstract (en)

The lack of donors is the biggest obstacle to the widespread use of hematopoietic stem cell transplantation. The establishment and improvement of new transplantation schemes have made haploid hematopoietic stem cell transplantation a clinical routine, benefiting a large number of patients with hematological diseases. Haploid donors have become the most important source of donors for allogeneic hematopoietic stem cell transplantation in China. This article focuses on the current situation and future development trends of haploid hematopoietic stem cell transplantation in China, in order to increase the understanding of clinical doctors on haploid hematopoietic stem cell transplantation.

异基因造血干细胞移植是血液恶性肿瘤最重要的根治手段之一[1]。北京大学血液病研究所团队采用重组人粒细胞集落刺激因子（G-CSF）和抗胸腺细胞球蛋白（ATG）联合诱导免疫耐受，成功克服人类白细胞抗原（HLA）不合的免疫屏障，推动单倍体造血干细胞移植（以下简称单倍体移植）广泛开展。本文就单倍体移植在我国开展的现状及未来发展趋势作一综述。

一、我国单倍体移植应用现状

2000年，北京大学血液病研究所通过G-CSF联合ATG诱导供受者免疫耐受，完成国内首例单倍体移植，并在2004年首次报告58例单倍体移植临床资料，所有患者均实现稳定植入，Ⅱ～Ⅳ度急性移植物抗宿主病（GVHD）发生率仅为37.9%，移植后2年无白血病生存率为67.2%[2]。随后通过完善植入不良防治、供者优选原则等多项关键移植技术，形成全球首个非体外去T细胞单倍体移植体系—“北京方案”。多中心、前瞻注册临床研究表明，基于“北京方案”的单倍体移植可以获得与全相合移植相似的临床疗效[3]。中国造血干细胞移植登记组资料显示，与美国后置环磷酰胺（PTCY）单倍体移植方案相比，“北京方案”在无进展生存率（74.3%对61%，*P*＝0.045）、总生存率（78.3%对65.2%，*P*＝0.039）和移植相关死亡率（12%对27.3%，*P*＝0.008）均具有明显优势[4]。

自2014年起，单倍体移植已经超越同胞相合移植成为我国首位造血干细胞移植模式[5]，移植例数从2008年的337例增长至2022年的8 170例，总例数超4.6万例，其中94%采用了“北京方案”移植模式。

二、单倍体移植体系的优化

（一）创新方案改善造血重建不良

北京大学血液病研究所团队发现骨髓微环境多种组分异常，尤其是骨髓血管内皮细胞数量和功能异常（例如活性氧水平异常增高）所致造血干细胞损伤是导致移植后造血重建不良的重要原因。基于移植前骨髓血管内皮细胞数量（<0.1%）可识别单倍体移植后植入功能不良（PGF）高危患者，口服活性氧清除剂乙酰半胱氨酸可显著降低PGF发生率[6]。

二次移植是PGF患者最重要的挽救治疗手段。北京大学血液病研究所团队采用新预处理方案（氟达拉滨30 mg·m^−2^·d^−1^，−6 d～−2 d；环磷酰胺1 g·m^−2^·d^−1^，−5 d、−4 d；巴利昔单抗20 mg/d，−1 d、+4 d）以及更换供者的二次移植，所有PGF患者在二次移植后均实现中性粒细胞植入，1年移植相关死亡率、总生存率分别为23.1%、56.6%[7]。此外，航天中心医院团队采用供者纯化CD34^+^细胞输注治疗单倍体移植后PGF获得良好效果[8]，但其有效性和安全性仍需大样本、前瞻性临床研究证实。

（二）多措并举降低GVHD

不同单倍体供者移植后GVHD的发生风险不一样，北京大学血液病研究所在G-CSF联合ATG基础上，联合移植后小剂量环磷酰胺，显著降低母亲、旁系供者单倍体移植后重度急性GVHD和非复发死亡发生率，提高无GVHD/无复发生存率[9]。

Chang等[10]发现基于骨髓采集物CD4^+^细胞和CD8^+^细胞比例这一生物标记可将单倍体移植患者划分为GVHD高危和低危，高危患者接受小剂量糖皮质激素强化预防可以将急性GVHD发生率从48%降低到21%。Shen等[11]通过机器学习建立了预测模型，可以有效预测单倍体外周血干细胞移植后重度急性GVHD的发生。也有研究探讨了细胞因子水平与单倍体移植后GVHD相关性，但不同研究间结果仍存争议。

北京大学血液病研究所发现糖酵解在急性GVHD中发挥重要作用，并据此设计糖皮质激素联合甲氨蝶呤作为急性GVHD一线治疗方案并获得良好效果，28天总有效率为87%[12]。此外，中国人民解放军总医院采用糖皮质激素联合芦可替尼方案一线治疗单倍体移植后急性GVHD，28天完全反应率为96.9%[13]。对于急性GVHD的二线治疗，真实世界研究证明巴利昔单抗治疗糖皮质激素耐药的急性GVHD有效、安全[14]。

（三）抢先干预降低移植后复发率

新型靶向药物维持治疗在移植后复发预防中发挥重要作用。对于FLT3阳性的急性髓系白血病，单倍体移植后索拉非尼维持治疗组2年累积复发率为17.5%，显著低于对照组的33.6%[15]。移植后可测量残留病（measurable residual disease, MRD）阳性的患者采用抢先干预是预防复发的另一重要手段。在中国真实世界研究中，接受干扰素α抢先干预的单倍体移植患者，2年MRD转阴率达到82.8%，复发率、无白血病生存率分别为15%、82.9%[16]。

（四）改良新方案拓展单倍体移植适应证

1. 单倍体移植治疗老年白血病：北京大学血液病研究所团队通过适当降低环磷酰胺剂量减轻预处理毒性，采用氟达拉滨加强免疫抑制，形成了适合老年患者的单倍体移植预处理方案，55岁以上血液肿瘤患者，单倍体移植后2年的移植相关死亡率、总生存率分别为23.3%、63.5%[17]。我国50岁以上allo-HSCT例数已从2019年的974例增加到2021年的2 950例，其中60岁以上患者例数从2019年的120例增加到2021年的506例，单倍体供者占比高达67%[18]。

2. 单倍体移植治疗非恶性血液病：北京大学血液病研究所建立适合重型再生障碍性贫血患者的新型单倍体移植预处理方案（白消安3.2 mg·kg^−1^·d^−1^×2 d、环磷酰胺50 mg·kg^−1^·d^−1^×4 d和ATG 2.5 mg·kg^−1^·d^−1^×4 d），使得单倍体移植后的无治疗失败生存率均与全相合移植非常接近，显著优于仅接受免疫抑制治疗的患者[19]。目前单倍体移植已经被中国指南推荐用于重型再生障碍性贫血患者的一线治疗，我国接受allo-HSCT的再生障碍性贫血患者中，单倍体移植占比超过50%，单倍体移植例数从2019年的648例增加到2021年的1 574例[5],[18]。

针对输血依赖的地中海贫血，在Hu等[20]的中国多中心研究中，采用PTCY预处理方案，单倍体移植后中性粒细胞和血小板植入率分别为96.3%、94.4%，总生存率、无事件生存率分别为98.1%、90.7%。我国接受allo-HSCT的地中海贫血患者中，单倍体移植占比从2019年的37%增加到2021年的50%，单倍体移植例数也从2019年的187例增加到2021年的660例。

三、新型免疫治疗助力单倍体移植更大的发展

嵌合抗原受体T（CAR-T）细胞治疗等新型免疫治疗靶向性强、短期缓解率高，但抗白血病的机制较单一，远期生存不尽人意。allo-HSCT可以通过多个途径发挥抗肿瘤作用，彻底清除残留的肿瘤细胞，实现持久的无病生存。单倍体移植与新型免疫治疗有机结合，可进一步提高移植的疗效和安全性。

（一）新型免疫治疗提高单倍体移植的疗效

1. CAR-T细胞治疗：对于难治/复发急性B淋巴细胞白血病患者，CAR-T桥接单倍体移植后2年的无事件生存率、总生存率、复发率分别为76.0%、84.3%、19.7%[21]；即使是CAR-T细胞治疗已经达到MRD阴性的完全缓解，桥接单倍体移植患者的复发率和无白血病生存率仍显著优于没有桥接移植的患者[22]。

也有研究探讨了供者来源CAR-T细胞在治疗allo-HSCT后复发中的作用。Zhao等[23]的前瞻性临床研究中，12例移植后MRD阳性的急性B淋巴细胞白血病患者（其中66.7%接受单倍体移植）接受供者来源CAR-T细胞治疗后全部实现MRD转阴，无论是复发率还是无白血病生存率均显著优于接受传统供者淋巴细胞输注的患者。但接受供者来源CD19 CAR-T细胞治疗移植后复发的急性B淋巴细胞白血病具有较高的缓解率，但肿瘤负荷较高的患者远期生存尚不能令人满意[24]。

2. 双特异衔接抗体（BiTE抗体）：浙江大学移植团队采用贝林妥欧单抗治疗移植后复发的急性淋巴细胞白血病，4例患者均获得完全缓解，其中3例达到MRD阴性[25]，但BiTE抗体如何更好地与单倍体移植结合还需进一步研究。

（二）新型免疫治疗提高单倍体移植的安全性

1. 病毒特异性细胞毒性T细胞（CTL）：巨细胞病毒（CMV）是移植后最常见的严重病毒感染。北京大学血液病研究所团队首次提出CMV-CTL过继回输后体内持久免疫恢复来源于内源性CMV特异性免疫重建[26]；随后的大宗队列研究进一步证实了CMV-CTL可安全、有效地治疗移植后CMV感染[27]。

2. 间充质干细胞：重庆新桥医院移植团队设计了联合使用间充质干细胞的慢性GVHD预防方案，发现间充质干细胞预防组慢性GVHD发生率显著低于对照组［27.4%（95% *CI* 16.2%～38.6%）对49.0%（95% *CI* 36.5%～61.5%）］[28]。随后的多中心、开放标签、随机对照研究进一步确认了该方案的有效性，间充质干细胞预防组、对照组慢性GVHD发生率分别为15.8%（95% *CI* 8.6%～26.6%）、26.2%（95% *CI* 16.8%～37.9%）（*P*＝0.04），无GVHD/无复发生存率分别为59.5%（95% *CI* 48.1%～69.9%）、26.2%（95% *CI* 17.6%～37.2%）（*P*＝0.04）[29]。

四、单倍体移植发展前景

单倍体移植仍有诸多热点问题亟待解决。例如，GVHD依然影响单倍体移植预后，通过进一步阐明单倍体移植的免疫耐受机制，将为预防和治疗GVHD提供理论基础。此外，通过促进移植后病毒特异性CTL重建，将为单倍体移植后难治性病毒感染的预防和治疗提供新思路。随着治疗新靶点的不断发现以及大量新型靶向药物、新型细胞治疗的研发，如何将这些治疗手段的短期缓解效果和单倍体移植的长期疾病控制能力有机结合，是改善难治/复发血液肿瘤疗效的关键。随着这些难题的不断被解决，单倍体移植必将迎来更大的发展，为更多的血液疾病患者带来根治的希望。

## References

[1] Lv M, Shen MZ, Mo XD (2023). Development of allogeneic hematopoietic stem cell transplantation in 2022: Regenerating “Groot” to heal the world[J]. Innovation (Camb).

[2] 黄 晓军, 韩 伟, 许 兰平 (2004). HLA配型不合情况下造血干细胞移植的新方法[J]. 北京大学学报(医学版).

[3] Wang Y, Liu QF, Xu LP (2015). Haploidentical vs identical-sibling transplant for AML in remission: a multicenter, prospective study[J]. Blood.

[4] Tang F, Xu Y, Chen H (2020). Comparison of the clinical outcomes of hematologic malignancies after myeloablative haploidentical transplantation with G-CSF/ATG and posttransplant cyclophosphamide: results from the Chinese Bone Marrow Transplantation Registry Group (CBMTRG)[J]. Sci China Life Sci.

[5] Xu LP, Lu PH, Wu DP (2021). Hematopoietic stem cell transplantation activity in China 2019: a report from the Chinese Blood and Marrow Transplantation Registry Group[J]. Bone Marrow Transplant.

[6] Wang Y, Kong Y, Zhao HY (2022). Prophylactic NAC promoted hematopoietic reconstitution by improving endothelial cells after haploidentical HSCT: a phase 3, open-label randomized trial[J]. BMC Med.

[7] Sun YQ, Wang Y, Wang FR (2021). Graft Failure in Patients With Hematological Malignancies: A Successful Salvage With a Second Transplantation From a Different Haploidentical Donor[J]. Front Med (Lausanne).

[8] 费 新红, 贺 俊宝, 程 昊钰 (2018). 纯化供者CD34+细胞输注治疗单倍型造血干细胞移植后移植物功能不良12例临床分析[J]. 中华血液学杂志.

[9] Wang Y, Wu DP, Liu QF (2019). Low-dose post-transplant cyclophosphamide and anti-thymocyte globulin as an effective strategy for GVHD prevention in haploidentical patients[J]. J Hematol Oncol.

[10] Chang YJ, Xu LP, Wang Y (2016). Controlled, randomized, open-label trial of risk-stratified corticosteroid prevention of acute graft-versus-host disease after haploidentical transplantation[J]. J Clin Oncol.

[11] Shen MZ, Hong SD, Lou R (2022). A comprehensive model to predict severe acute graft-versus-host disease in acute leukemia patients after haploidentical hematopoietic stem cell transplantation[J]. Exp Hematol Oncol.

[12] Xu Z, Mo X, Kong Y (2023). Mini-dose methotrexate combined with methylprednisolone as a first-line treatment for acute graft-versus-host disease: A phase 2 trial[J]. J Transl Int Med.

[13] Hou C, Dou L, Jia M (2021). Ruxolitinib combined with corticosteroids as first-line therapy for acute graft-versus-host disease in haploidentical peripheral blood stem cell transplantation recipients[J]. Transplant Cell Ther.

[14] Mo XD, Hong SD, Zhao YL (2022). Basiliximab for steroid-refractory acute graft-versus-host disease: A real-world analysis[J]. Am J Hematol.

[15] Xuan L, Wang Y, Huang F (2020). Sorafenib maintenance in patients with FLT3-ITD acute myeloid leukaemia undergoing allogeneic haematopoietic stem-cell transplantation: an open-label, multicentre, randomised phase 3 trial[J]. Lancet Oncol.

[16] Fan S, Pan TZ, Dou LP (2023). Preemptive interferon-α therapy could prevent relapse of acute myeloid leukemia following allogeneic hematopoietic stem cell transplantation: A real-world analysis[J]. Front Immunol.

[17] Sun YQ, Han TT, Wang Y (2021). Haploidentical Stem Cell Transplantation With a Novel Conditioning Regimen in Older Patients: A Prospective Single-Arm Phase 2 Study[J]. Front Oncol.

[18] Xu LP, Lu DP, Wu DP (2023). Hematopoietic Stem Cell Transplantation Activity in China 2020-2021 During the SARS-CoV-2 Pandemic: A Report From the Chinese Blood and Marrow Transplantation Registry Group[J]. Transplant Cell Ther.

[19] Xu ZL, Zhou M, Jia JS (2019). Immunosuppressive therapy versus haploidentical transplantation in adults with acquired severe aplastic anemia[J]. Bone Marrow Transplant.

[20] Hu J, Gong S, Chen K (2023). Haploidentical transplant for paediatric patients with severe thalassaemia using post-transplant cyclophosphamide and methotrexate: A prospectively registered multicentre trial from the Bone Marrow Failure Working Group of Hunan Province, China[J]. Br J Haematol.

[21] Hu GH, Zhao XY, Zuo YX (2021). Unmanipulated haploidentical hematopoietic stem cell transplantation is an excellent option for children and young adult relapsed/refractory Philadelphia chromosome-negative B-cell acute lymphoblastic leukemia after CAR-T-cell therapy[J]. Leukemia.

[22] Zhao H, Wei J, Wei G (2020). Pre-transplant MRD negativity predicts favorable outcomes of CAR-T therapy followed by haploidentical HSCT for relapsed/refractory acute lymphoblastic leukemia: a multi-center retrospective study[J]. J Hematol Oncol.

[23] Zhao XY, Xu ZL, Mo XD (2022). Preemptive donor-derived anti-CD19 CAR T-cell infusion showed a promising anti-leukemia effect against relapse in MRD-positive B-ALL after allogeneic hematopoietic stem cell transplantation[J]. Leukemia.

[24] Chen YH, Zhang X, Cheng YF (2020). Long-term follow-up of CD19 chimeric antigen receptor T-cell therapy for relapsed/refractory acute lymphoblastic leukemia after allogeneic hematopoietic stem cell transplantation[J]. Cytotherapy.

[25] Wu H, Cai Z, Shi J (2021). Blinatumomab for HLA loss relapse after haploidentical hematopoietic stem cell transplantation[J]. Am J Cancer Res.

[26] Zhao XY, Pei XY, Chang YJ (2020). First-line therapy with donor-derived human cytomegalovirus (HCMV)-specific T cells reduces persistent HCMV infection by promoting antiviral immunity after allogenic stem cell transplantation[J]. Clin Infect Dis.

[27] Pei XY, Zhao XY, Liu XF (2022). Adoptive therapy with cytomegalovirus-specific T cells for cytomegalovirus infection after haploidentical stem cell transplantation and factors affecting efficacy[J]. Am J Hematol.

[28] Gao L, Zhang Y, Hu B (2016). Phase II multicenter, randomized, double-blind controlled study of efficacy and safety of umbilical cord-derived mesenchymal stromal cells in the prophylaxis of chronic graft-versus-host disease after HLA-haploidentical stem-cell transplantation[J]. J Clin Oncol.

[29] Huang R, Chen T, Wang S (2024). Mesenchymal Stem Cells for Prophylaxis of Chronic Graft-vs-Host Disease After Haploidentical Hematopoietic Stem Cell Transplant: An Open-Label Randomized Clinical Trial[J]. JAMA Oncol.

